# Physicochemical Characterization, Antioxidant Activity, and Phenolic Compounds of Hawthorn (*Crataegus* spp.) Fruits Species for Potential Use in Food Applications

**DOI:** 10.3390/foods9040436

**Published:** 2020-04-04

**Authors:** Abolfazl Alirezalu, Nima Ahmadi, Peyman Salehi, Ali Sonboli, Kazem Alirezalu, Amin Mousavi Khaneghah, Francisco J. Barba, Paulo E.S. Munekata, Jose M. Lorenzo

**Affiliations:** 1 Department of Horticultural Sciences, Faculty of Agriculture, Urmia University, Urmia 5756151818, Iran; a.alirezalu@urmia.ac.ir; 2 Department of Horticultural Sciences, Faculty of Agriculture, Tarbiat Modares University, Tehran 1411713116, Iran; 3 Medicinal Plants and Drugs Research Institute, Shahid Beheshti University, Tehran 1983969411, Iran; p-salehi@sbu.ac.ir (P.S.); a-sonboli@sbu.ac.ir (A.S.); 4 Department of Food Science and Technology, Ahar Faculty of Agriculture and Natural Resources, University of Tabriz, Tabriz, Iran; kazem.alirezalu@tabrizu.ac.ir; 5 Department of Food Science, Faculty of Food Engineering, University of Campinas (UNICAMP), Campinas, 13083-862 São Paulo, Brazil; mousavi@unicamp.br; 6 Nutrition and Food Science Area, Preventive Medicine and Public Health, Food Science, Toxicology and Forensic Medicine Department, Faculty of Pharmacy, Universitat de València, Avda.Vicent Andrés Estellés, s/n, 46100 Burjassot, València, Spain; francisco.barba@uv.es; 7 Centro Tecnológico de la Carne de Galicia, rúa Galicia n° 4, Parque Tecnológico de Galicia, San Cibrao das Viñas, 32900 Ourense, Spain; paulosichetti@ceteca.net

**Keywords:** *Crataegus* spp., bioactive compounds, physicochemical characteristics, phenolic compounds, flavonoids

## Abstract

Hawthorn belongs to the *Crataegus* genus of the Rosaceae family and is an important medicinal plant. Due to its beneficial effects on the cardiovascular system and its antioxidant and antimicrobial activity hawthorn has recently become quite a popular herbal medicine in phytotherapy and food applications. In this study, physicochemical characterization (color parameters, pH, titratable acidity, total soluble solids, soluble carbohydrate, total carotenoid, total phenols, and flavonoid contents), antioxidant activity (by ferric-reducing antioxidant power, FRAP assay), and quantification of some individual phenolic compounds of fruits of 15 samples of different hawthorn species (*Crataegus* spp.) collected from different regions of Iran were investigated. According to findings, the total phenols, total flavonoid content, and antioxidant activity were in the range of 21.19–69.12 mg gallic acid equivalent (GAE)/g dry weight (dw), 2.44–6.08 mg quercetin equivalent (QUE)/g dw and 0.32–1.84 mmol Fe^++^/g dw, respectively. Hyperoside (0.87–2.94 mg/g dw), chlorogenic acid (0.06–1.16 mg/g dw), and isoquercetin (0.24–1.59 mg/g dw) were found to be the most abundant phenolic compounds in the extracts of hawthorn fruits. The considerable variations in the antioxidant activity and phenolic compounds of hawthorn species were demonstrated by our results. Hence, the evaluation of hawthorn genetic resources could supply precious data for screening genotypes with high bioactive contents for producing natural antioxidants and other phytochemical compounds valuable for food and pharma industries.

## 1. Introduction

Hawthorn berry (*Crataegus* L.) is a genus of over 1000 species, belonging to the subfamily Maloideae in the Rosaceae family, widely distributed in Asia and Europe [1]. Hawthorn fruits contain high amounts of phenolic compounds, which are used as medicinal remedies with a variety of biological activities such as antitumor [2], antispasmodic, cardiotonic, diuretic, hypotensive, anti-atherosclerotic [3], and anti-inflammation [4]. Several studies have shown that extracts of hawthorn fruit offer beneficial effects on the heart and also for blood circulation [5]. The antioxidant and antimicrobial effects of phenolic compounds have been investigated among various commercial food products such as lamb burgers [6,7], frankfurter-type sausages [8], and pig liver pâté [9].

Polyphenols, bioflavonoids, flavonoid glycosides, triterpenoids, oligomeric procyanidins, antioxidants, vitamins, tannins, organic acids, and some phenolic acids are the main active constituents of the *Crataegus* species [10,11,12]. The fruits of different *Crataegus* species could be considered as a rich source of antioxidants, due to their high phenolic compositions and some well-known antioxidant compounds namely, hyperoside, isoquercetin, epicatechin, chlorogenic acid, quercetin, rutin, and protocatechuic acids [13,14,15].

Environmental conditions, postharvest handling, and processing are among the factors that might have an influence on the physical characteristics, chemical composition of phenolic compounds, and their antioxidant activity [16,17,18]. In addition, the amount of bioactive compounds such as flavonoids and phenolic acids is also affected by genetic variation among species, within the same species, and maturity of plant organs at its harvest [19]. The variation in physicochemical characteristics, phytochemicals, and antioxidant activity among different species of *Crataegus* revealed were well documented by previous studies [15,17,20,21,22,23].

Although synthetic antioxidants and antimicrobial agents can effectively be used in food processing due to high stability and efficiency and low cost, there are significant concerns related to their potential health risks and toxicological aspects [24]. Therefore, some research has been performed to evaluate the performance of natural antioxidants and antimicrobials such as essential oils and plant extracts as alternatives to synthetic antioxidants [25,26,27,28,29]. On the other hand, limited sources of natural antioxidants and antimicrobials and high price as well as shortage of new sources of safe and inexpensive antioxidants and antimicrobials of natural origin could be a plausible reason for the food and pharmaceutical industries to use synthetic antioxidants instead [30,31]. Thus, there is a growing interest in using natural compounds and their application in food, nutrition, and medical treatments [32,33,34,35,36]. Iran is known as one of the primary centers of genetic diversity of *Crataegus*; however, few studies have been carried out on the content of total phenolic compounds, antioxidant activity, and antimicrobial effects of this genus in Iran. In this regard, the current investigation was devoted to assess the physicochemical characterization (color parameters, pH, titratable acidity, total soluble solids, soluble carbohydrate, total carotenoid, total phenols, and flavonoid contents), antioxidant activity, and also quantification of some individual phenolic compounds of fruits of 15 samples of different hawthorn species (*Crataegus* spp.) collected from different regions of Iran.

## 2. Materials and Methods 

### 2.1. Plant Sample Collection

**Figure 1:**
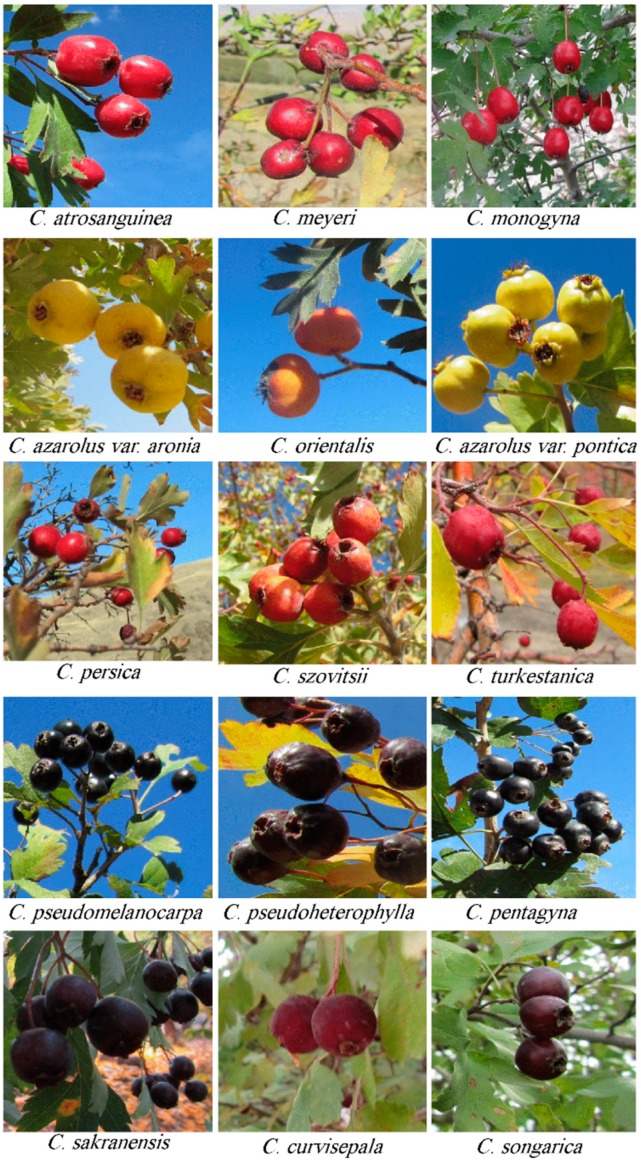
Fruits of different hawthorn (*Crataegus*) species.

A total of 15 fruit specimens (Figure 1) were collected from wild-growing *Crataegus* species from 7 provinces of Iran (Table 1), during 2015. The samples were transferred to the laboratory and physicochemical characteristics were measured in the shortest time possible.

### 2.2. Preparation of Fruit Extracts

Fruits of each species were dried using convection oven at 45±2 °C for 24 h and ground to homogenize particle size before extraction. Powdered samples (1 g) were extracted by ultrasound (for 30 min at 25 °C) using methanol/water (80:20, 25 mL), then they were filtered.

### 2.3. Physicochemical Characterization

Total soluble solids (TSS), expressed as % malic acid, of fruits were measured by a handheld refractometer (model 9703, Japan) and titratable acidity (TA) by titration of fruit juice with 0.1 N NaOH to pH 8.3 and data were expressed as a percentage of malic acid. Juice pH of fruit samples was measured using a pH meter (Model 744, Metrohm). Water-soluble carbohydrate contents (TSC) of fruit samples were measured using the anthrone method [37]. Total carotenoids were extracted by acetone and measured by the spectrophotometric method. Absorbance at 662, 645, and 470 nm was used to determine their concentrations [38]. The color parameters of fruits such as a* (redness/greenness), b* (yellowness/blueness), and L* (whiteness/darkness) were measured by Hunter Lab (Hunter Associates Laboratory, VA, USA). Chroma (*C*) and hue (*h*°) were also calculated from a* and b* coordinates.

### 2.4. Total Phenol Content (TPC)

TPC was assayed according to Singleton et al. [39]. The extracted samples (0.5 mL of different dilutions) were mixed with Folin–Ciocalteu reagent (5 mL, 1:10 diluted with distilled water) for 5 min and aqueous Na_2_CO_3_ (4 mL, 1 M) was then added. The mixture was allowed to stand for 15 min, and the phenols were determined by spectrophotometer at 765 nm. The standard curve was prepared by 0, 50, 100, 150, 200, and 250 mg mL^−1^ solutions of gallic acid in methanol: water (50:50, *v/v*). Total phenols values are expressed in terms of gallic acid equivalent (mg GAE/g dry weight fruit), which is a common reference compound. 

### 2.5. Total Flavonoid Content (TFC)

TFC of the fruits extracts was determined using the aluminum chloride colorimetric method with slight modification using quercetin as standard and the results were expressed as mg of quercetin equivalents per g dry weight of the plant (mg QUE/g dw). Briefly, the extract solution (0.5 mL) was mixed with 1.5 mL of 80% methanol, 0.1 mL of 10% aluminum chloride hexahydrate (AlCl_3_), 0.1 mL of 1 M potassium acetate (CH_3_COOK), and 2.8 mL of deionized water. After incubation at room temperature for 30 min, the absorbance of the reaction mixture was measured at 415 nm against deionized water blank [40].

### 2.6. Antioxidant Activity

The antioxidant activity of hawthorn fruit extracts was calculated using ferric-reducing antioxidant power (FRAP) assay. Diluted extracts from different organs of hawthorn (100 µL) and 3.0 mL of freshly prepared FRAP-reagent (containing 25 mL of 300 mM acetate buffer, pH 3.6 plus 2.5 mL of 10 mM tripyridyltriazine stock solution in 40 mM HCl plus 2.5 mL of 20 mM FeCl_3_·6H_2_O) were mixed. The absorbance was recorded at 593 nm against a blank, containing 100 µL of resembling solvent, after 30 min incubation at 37 °C. The FRAP-value was calculated from the calibration curve of FeSO_4_·7H_2_O standard solutions, covering the concentration ranging 100–1000 µmol/L and expressed as mmol Fe^++^/g dry weight plant [13].

### 2.7. Preparation of Standard Solutions

One milligram of a standard of each phenolic compound (chlorogenic acid, vitexin 2-O-rhamnoside, vitexin, rutin, hyperoside, isoquercetin, and quercetin; from Sigma, US) was weighed accurately and dissolved in 1:1 MeOH/water in a 10 mL volumetric flask to prepare the stock solution. For calibration curves, the stock solution was diluted with 1:4 MeOH/water to obtain the concentration sequence. Ten microliters of each solution was injected into HPLC. The linear range and the equations of linear regression were obtained through a sequence of 1000, 500, 250, 100, 50, 20, 10, 5, 2, and 1 mg/L. Mean areas (*n* = 3) generated from the standard solutions were plotted against concentration to establish calibration equations.

### 2.8. Quantification of Phenolic Compounds 

Quantification of some individual phenolic compounds (i.e., chlorogenic acid, vitexin 2"-*O*-rhamnoside, vitexin, rutin, hyperoside, quercetin, and isoquercetin) by high-pressure liquid chromatography (HPLC) was carried out using a Knauer HPLC apparatus consisting of a 1000 Smartline pump, a 5000 Smartline manager solvent organizer, and a 2800 Smartline photo-diode array detector. The injection was carried out through a 3900 Smartline autosampler injector equipped with a 100 µL loop. The temperature control of the column was made with a jet stream 2 plus oven (Knauer, advanced scientific instrument, Berlin, Germany). The separation was achieved on an Eclipse XDB-C18 (4.6 mm × 250 mm, 5 µm), Agilent (USA) column. Data acquisition and integration were performed with EZChrome Elite software. The flow rate of the mobile phase was kept at 1 mL/min. Solvent A was water containing formic acid (0.05%), and Solvent B was acetonitrile/methanol (80:20, v/v). The gradient conditions were as follows: 0–5 min, 10% B; 5–15 min, 10–18% B; 15–25 min, 18% B; 25–30 min, 18–25% B; 30–35 min, 25% B; 35–40 min, 25–35% B; 40–45 min, 35–60% B; 45–50 min 60–10% B, and 50–55 min with 10% B. The temperature of the column was kept at 25 °C. The partial loop injection volume was 10 µL. The detection wavelengths of the DAD (diode array detectors) were set at three selected positions: 320, 335, and 360 nm [41].

### 2.9. Statistical Analysis

SAS 9.1.3 software package (v.9, SAS Institute, USA) was used for statistical analysis of the data. All of the analyses were done in triplicate with an experiment in a completely randomized design. The Duncan test was used to compare pairs of means and determine statistical significance at the (*P* < 0.05) level. Furthermore, hierarchical cluster analysis (HCA) and principal component analysis (PCA) was performed among the variables analyzed using Minitab software. Heat-maps were used to visualize phenolic compounds in each species using GraphPad Prism software.

## 3. Results and Discussion

### 3.1. Physicochemical Characterization

The fruit’s external color was significantly variable amongst the different species of *Crataegus* (*P* < 0.001). The color range of hawthorn fruits is varied from yellow to black (yellow, yellow–orange, red, orange–red, purple, purple–black, and black). The highest a^*^ value (40.63) was obtained from *Crataegus atrosanguinea* species, while b^*^ values (56.93), L^*^ value (37.36), *C* (61.15), *h*° (77.78), were highest in the extracts of *Crataegus azarolus* var. *aronia*. The color characteristics of *Crataegus* spp. fruits are given in Table 2.

Results demonstrated that origin of species had significant effects (*P* < 0.001) regarding the chemical characteristics (pH, titratable acidity, total soluble solids, total soluble carbohydrate, and total carotenoid content) of hawthorn fruits (Table 3). The pH level of fruits was recorded in the range of 3.03–4.35. The pH was at its highest value in *Crataegus curvisepala*, whereas the lowest level correlated with *Crataegus orientalis*. The highest levels of acidity (TA) were observed in yellow fruits of hawthorn. The highest (1.17%) and lowest (0.75%) TA were obtained from *Crataegus azarolus* var. *pontica* and *Crataegus pentagyna* species, respectively. The highest total soluble solids (TSS) of fruits (23.43 °Brix) was found in C. *azarolus* var. *pontica*, and the lowest (14.99 °Brix) occurred in *C. pentagyna*. The total soluble carbohydrate was at its highest value (19.43%) in *C. azarolus* var. *aronia*, whereas the lowest level (5.27%) was found in *Crataegus monogyna*. The highest total carotenoid content (405.79 μg/g fruit weight) was obtained from C. *azarolus* var. *pontica* species.

The physicochemical characteristics of fruits are important indicators of their quality and maturation; key factors for achievement of market demands that have encouraged many researchers under different conditions overseas. These traits can be used for the evaluation of diversity and release of a new cultivar. Therefore, some investigations suggested that the physicochemical characteristics of hawthorn fruit are influenced by species and collection location (origin) [17,42,43,44]. 

In this regard, Li et al. [44] reported average L*, a*, b*, TSS, and TA values of hawthorn samples as from 33 to 54, from −5 to 17, from 12 to 21, 7%, and 3%, respectively, which are in agreement with our present results. On the other hand, Özcan et al. [17] showed average pH and acidity values of hawthorn fruits as 3.38% and 1.98%, respectively. Finally, Serçe et al. [21] noticed average soluble solids (%), pH, acidity (%), L*, a*, b*, c*, and h values of hawthorn samples as 16.5%, 3.19, 1.49%, 71.3, 1.8, 49.4, 51.4, and 87.2, respectively, which are in agreement with our present results. 

### 3.2. Total Phenol Content (TPC)

The TPC of fruits of hawthorn species is presented in Table 4. The amount of TPC of hawthorn fruits was significantly variable (*P* < 0.001) among species, ranging from 21.19 to 69.12 mg GAE/g dry weight.

Total phenol content was at its highest value in the fruits of *C. pentagyna*, whereas the lowest level was found in the fruits of *Crataegus turkestanica*. According to results, TPC can be significantly influenced by both the species and also the sampling location. Accordingly, some studies proposed that the polyphenolic content of plant organs is influenced by genotype and habitat conditions [43], and moreover, altitude, light, temperature, and content of nutritive matter available in the soil may influence phenylpropanoid metabolism [45]. The time of harvesting (the stage of maturity) is also can be accounted as a very important factor. Similar findings were also obtained in term of the total phenol content i.e., 2.9 mg GAE/g dw for *Crataegus pinnatifida* [46], and 26.4 mg GAE/g dw for *C. monogyna* [47]. In another study, the total content of polyphenols in fruits of *C. pinnatifida* was 96.9 ± 4.3 mg gallic acid equivalents/g weight [48]. The health and technological benefits associated with plant compounds in value-added food products had been attributed to the antioxidant and antimicrobial activity of phenols content [49,50]. 

### 3.3. Total Flavonoid Content (TFC)

Table 4 shows the TFC in fruits of hawthorn. The content of total flavonoids was significantly variable (*P* < 0.001) among species and ranging from 2.44 to 6.08 mg QUE/g dw. Total flavonoid content was highest in *Crataegus meyeri*, whereas the lowest level was found in the fruits of *Crataegus szovitsii*. The TFC is influenced by the interaction between species and sampling location. Furthermore, environmental factors have a significant contribution to the total flavonoid content in plants. The total flavonoid content found in the present study was similar to those reported for other hawthorn species in previous studies, i.e., 1.47 mg/g dw for *C. monogyna* fruits [51], 23.68 mg/g dw for *C. pentagyna* fruits [52], and 0.81 mg/g dw for *C. azarolus* fruits [53]. 

### 3.4. Antioxidant Activity

The antioxidant activity was widely varied (*P* < 0.001) in species of *Crataegus,* ranging from 0.32–1.84 mmol Fe^++^/g dw (Table 4). The highest antioxidant activity was observed in the fruits of *C. pentagyna*, whereas the lowest activity was found in the fruits of *Crataegus persica*. The evaluation of antioxidant activity of *Crataegus* species demonstrated that they could possess considerable antioxidant activities due to the presence of polyphenolic compounds. Moreover, the total and individual phenolic compounds are the main responsible agents for the antioxidant activity of hawthorn fruits. Among them, chlorogenic acid, hyperoside, rutin, spiraeoside, quercetin 3-glucoside (isoquercetin), quercetin, (-)-epicatechin, and procyanidin B2 were suggested to be the compounds with strong radical-scavenging activity in floral bud extracts of hawthorn [54]. The ethanol extract of *C. monogyna* fruits contained higher levels of phenolic compounds and showed greater radical-scavenging activities than the aqueous extract of the fruits [47]. Most of the reports regarding antioxidant activity of *Crataegus* species were correlated with either fruits, aerial parts, or flowers of the plant [55]. 

Oxidation can affect the sensory attributes, nutritional value, texture properties, and shelf life stability of food by decomposition of proteins, vitamins, unsaturated essential fatty acids, and pigments such as anthocyanin, carotenoid, and myoglobin [56]. A significant relation between phenol content and antioxidant activity of plant constituents has been reported by Agregan et al [57] and Roselló-Soto et al. [58]. In addition, some studies have revealed that plant compounds are a natural antioxidant that can significantly reduce lipid oxidation, for example, in ground beef with four garlic-derived compounds [59], pork patties with natural antioxidant [60], rabbit meat with oregano, rosemary, and vitamin E [61], roast beef patties with chili pepper extract [62], frankfurter-type sausage with combined effect of mixed plant extracts (green tea, stinging nettle, and olive leaves extracts) and natural antimicrobial agents [8]. 

### 3.5. Quantification of Phenolic Compounds

**Figure 2:**
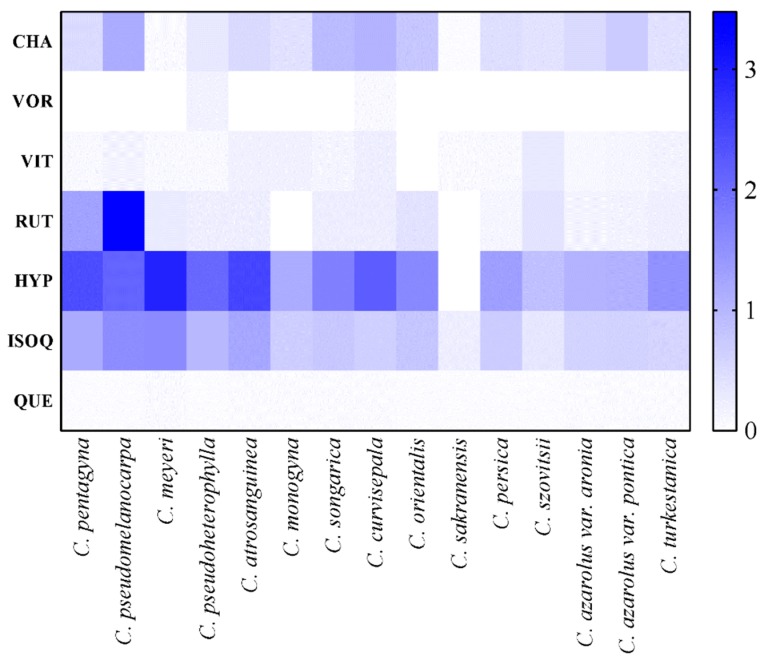
Phenolic compound distribution in fruits of hawthorn species with heat map visualization. From white for low concentrations to blue for high concentrations (mg/g DW). CHA, chlorogenic acid; VOR, vitexin 2”-O-rhamnoside; VIT, vitexin; RUT, rutin; HYP, hyperoside; ISOQ, isoquercetin; and QUE, quercetin.

Table 5 summarized the proximate composition of phenolic compounds in all the 15 species analyzed. The amounts of phenolic compounds were significantly variable amongst different species (*P* < 0.001). In this regard, hyperoside, chlorogenic acid, and isoquercetin were found to be the most abundant phenolic compounds in the extracts of hawthorn fruits. However, in most species, vitexin 2"-*O*-rhamnoside was not detected and the quercetin content was very low. The heat map in Figure 2 can aid us to summarize quantitative data regarding the phenolic compound distribution in fruits of hawthorn species. Color was associated with the content of phenolic compounds: from white for low concentrations to blue for high concentrations. *Crataegus pseudomelanocarpa* had the highest level (1.16 mg/g dw) of chlorogenic acid, and *C. meyeri* had the lowest level (0.06 mg/g dw) among the fruits of the studied species. *Crataegus pseudoheterophylla* had the highest content (0.17 mg/g dw) of vitexin 2-*O*-rhamnoside among the species studied. In most species, vitexin 2-*O*-rhamnoside was not detected. Vitexin was in the highest value (0.31 mg/g dw) in *C. szovitsii* whereas the lowest level (0.06 mg/g dw) was found in *Crataegus sakranensis* among the fruits of the studied species. *C. pseudomelanocarpa* had the highest level (2.68 mg/g dw) of rutin among the fruits of the studied species.

Hyperoside and isoquercetin were at the highest value (2.94 and 1.59 mg/g dw), respectively, in the *C. meyeri* species. The present study showed that total and individual phenolic compounds are the main contributor to the antioxidant activity of hawthorn fruits, which also can be influenced by the variation of fruits species. Moreover, several environmental factors affect the concentration of phenolic compounds in plants [63]. In this context, it was reported that higher growing temperatures and level of CO_2_ increase flavonoid content and concentrations of the phenolic compounds [64]. Furthermore, soil conditions affect plant phenolic composition. Soil fertilization (such as high level of nitrogen) and increase in soil moisture deficit led to the lower synthesis and hence lower levels of some certain phenolics [65]. Moreover, light stimulates the synthesis of phenolic compounds such as flavonoids, flavones, anthocyanins, and also PAL (phenylalanine ammonia-lyase) enzyme.

In general, variability in the reported phenolic compound contents and flavonoid concentrations within one species could be mainly related to differences in growth conditions [45], genetic background [66], and methodological differences [67].

### 3.6. Hierarchical Cluster Analysis and Principal Component Analysis

**Figure 3:**
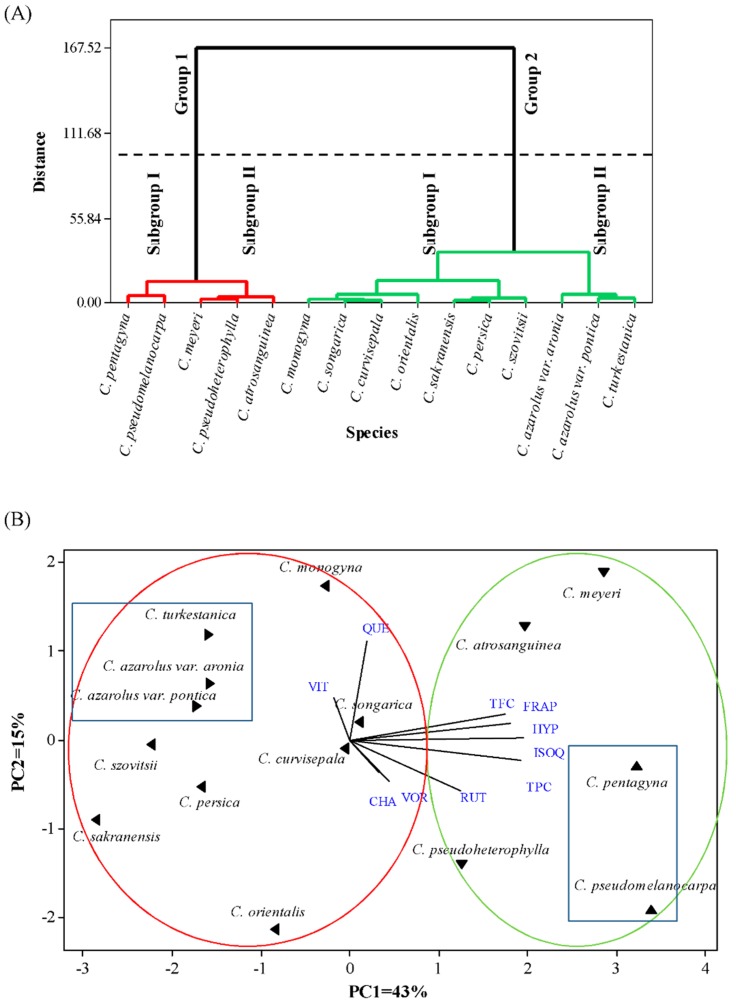
(**A**) Hierarchical cluster analysis (HCA) and (**B**) principal component analysis (PCA) of hawthorn species based on the 10 main traits: (TPC: total phenol content, TFC: total flavonoid content, FRAP: ferric-reducing antioxidant power (antioxidant activity), CHA: chlorogenic acid, VOR: vitexin 2-O-rhamnoside, VIT: vitexin, RUT: rutin, HYP: hyperoside, ISOQ: isoquercetin, and QUE: quercetin).

To evaluate the relationships and likely similarities among *Crataegus* species studied, hierarchical cluster analysis (HCA) was performed based on the 10 main traits (TPC, TFC, FRAP, CHA, VOR, VIT, RUT, HYP, ISOQ, and QUE). The cluster analysis was carried out by the Ward linkage method for agglomeration and the Euclidean distance as the criterion of proximity (Figure 3A). The resulting dendrogram had two major groups based on similarity. Each group was also divided into two subgroups. The first association consisted of five species (*C. pentagyna*, *C. pseudomelanocarpa*, *C. atrosanguinea*, *C. pseudoheterophylla*, and *C. meyeri*,) with high TPC, TFC, HYP, ISOQ, and antioxidant activity of fruit. It was shown that subgroup 1 species (*C. pentagyna* and *C. pseudomelanocarpa*) have higher RUT compound than the subgroup 2 species. The second association consisted of ten species with medium and low TPC, TFC, antioxidant activity, and other phenolic compounds of fruit. It was shown that subgroup 2 species (*C. azarolus* var. *aronia, C. azarolus* var. *pontica*, and *C. turkestanica*) have the lowest TPC compared to subgroup 1 and other species.

Principal component analysis (PCA) was applied, in order to classify the species studied according to the traits described above. PCA classification confirmed the results of cluster analysis (Figure 3B). A PCA was performed, reducing the multidimensional structure of the data and providing a two-dimensional map to explain the variance observed. The first two components of the PCA explained 58% of the total variance (43% for component 1 and 15% for component 2). The first component (PC1) was highly positively correlated with TPC, TFC, FRAP, HYP, ISOQ, and RUT. The second principal component (PC2) separated the samples according to CHA, VOR, VIT, and QUE compounds. 

## 4. Conclusions

The results of the present study demonstrated that total and individual phenolic compounds are the main contributor to the antioxidant activity of hawthorn fruits. Hyperoside, chlorogenic acid, and isoquercetin were found to be the most abundant phenolic compounds in the extracts of hawthorn fruits. To the best of our knowledge, this is the first report regarding antioxidant activity and determination of phenolic compounds (chlorogenic acid, vitexin 2"-*O*-rhamnoside, vitexin, rutin, hyperoside, quercetin, and isoquercetin) in fruits of *Crataegus* species grown in Iran. The fruits of different *Crataegus* species (especially *C. pseudomelanocarpa* and *C. pentagyna*) showed a high level of total phenol content as well as antioxidant activity. As a conclusion, our results clearly demonstrate the considerable variation in the antioxidant activity and phenolic compounds of hawthorn species. Hence, the evaluation of hawthorn genetic resources could supply precious data for screening genotypes with high bioactive contents for producing natural antioxidants and other phytochemical compounds valuable for food and pharma industries.

## Figures and Tables

**Table 1:** Sampling locations of the different *Crataegus* species studied.

Province	Species	Height	Latitude	Longitude
Semnan	*C. pentagyna*	1540	36° 02′N	53° 28′E
Mazandaran	*C. monogyna*	1081	36° 25′N	51° 52′E
Mazandaran	*C. pseudomelanocarpa*	1371	36° 23′N	51° 32′E
Mazandaran	*C. songarica*	1123	36° 25′N	51° 31′E
Bakhtiari	*C. azarolus* var. *aronia*	1913	31° 33′N	51° 12′E
Alborz	*C. azarolus* var. *pontica*	1846	36° 09′N	50° 42′E
East Azerbaijan	*C. sakranensis*	1694	38° 14′N	45° 42′E
East Azerbaijan	*C. turkestanica*	1690	38° 14′N	45° 42′E
East Azerbaijan	*C. meyeri*	1281	38° 49′N	47° 03′E
East Azerbaijan	*C. orientalis*	1277	38° 49′N	47° 03′E
East Azerbaijan	*C. curvisepala*	1196	38° 50′N	47° 02′E
Kurdistan	*C. atrosanguinea*	1633	35° 23′N	46° 55′E
Kurdistan	*C. persica*	1637	35° 23′N	46° 55′E
Kurdistan	*C. szovitsii*	1506	36° 06′N	46° 20′E
West Azerbaijan	*C. pseudoheterophylla*	1488	37° 27′N	44° 56′E

**Table 2:** Color parameters in fruits of different hawthorn (*Crataegus* spp.) species.

Species	Color parameters				
	a*	b*	L*	*C*	*h*°
*C. pentagyna*	0.36 ± 0.12^h^	0.14 ± 0.06^j^	0.17 ± 0.09^h^	0.39 ± 0.10^j^	18.98 ± 1.13^fg^
*C. monogyna*	33.95 ± 1.49^bc^	12.55 ± 1.30^fg^	7.37 ± 0.11^e^	36.21 ± 1.34^e^	20.18 ± 1.51^f^
*C. pseudomelanocarpa*	0.42 ± 0.23^h^	0.10 ± 0.13^j^	0.15 ± 0.02^h^	0.43 ± 0.11^j^	12.95 ± 0.34^i^
*C. songarica*	6.59 ± 1.29g	1.62 ± 0.15^j^	1.03 ± 0.32^h^	6.79 ± 0.27^i^	13.83 ± 1.76^i^
*C. azarolus* var. *aronia*	12.33 ± 0.19^f^	56.93 ± 1.17^a^	37.36 ± 1.97^a^	58.25 ± 1.29^a^	77.78 ± 1.21^a^
*C. azarolus* var. *pontica*	22.85 ± 1.82^e^	56.71 ± 1.43^a^	35.44 ± 1.87^a^	61.15 ± 1.55^a^	68.03 ± 1.85^b^
*C. sakranensis*	7.07 ± 0.46^g^	1.73 ± 0.07^j^	1.10 ± 0.27^h^	7.28 ± 0.38^i^	13.72 ± 1.65^i^
*C. turkestanica*	39.43 ± 1.67^a^	18.79 ± 1.34^e^	10.98 ± 1.29^d^	43.68 ± 1.98^cd^	25.45 ± 0.45^e^
*C. meyeri*	22.23 ± 1.36^e^	5.68 ± 0.19^i^	3.41 ± 0.13^g^	22.95 ± 1.83^h^	14.32 ± 0.92^hi^
*C. orientalis*	27.74 ± 1.76^d^	42.73 ± 1.92^b^	26.89 ± 1.32^b^	50.97 ± 1.87^b^	57.06 ± 0.26^c^
*C. curvisepala*	31.45 ± 1.43^c^	9.67 ± 0.39^gh^	5.70 ± 0.32^ef^	32.91 ± 1.10^f^	17.04 ± 1.18^gh^
*C. atrosanguinea*	40.63 ± 1.27^a^	23.53 ± 1.20^c^	13.74 ± 1.10^c^	46.96 ± 1.29^b^	30.04 ± 0.87^d^
*C. persica*	39.49 ± 1.45^a^	22.22 ± 1.49^cd^	12.98 ± 1.37^cd^	45.31 ± 1.19^bc^	29.36 ± 1.35^d^
*C. szovitsii*	36.01 ± 2.07^b^	19.53 ± 1.59^de^	11.60 ± 1.15^d^	40.99 ± 1.72^d^	28.46 ± 1.13^d^
*C. pseudoheterophylla*	26.40 ± 1.13^d^	7.07 ± 0.27^hi^	4.19 ± 0.23^fg^	27.33 ± 1.20^g^	14.99 ± 0.24^hi^
Significant level	***	***	***	***	***

**Note:** a*, redness/greenness index; b*, yellowness/blueness index; L*, lightness/darkness index; C, chroma index; and *h*°, hue index. *** Significant at 0.1% level, means with different letters are statistically significant at a 5% level of probability.

**Table 3:** Physicochemical characteristics of hawthorn (*Crataegus* spp.) species.

Species	pH	TA ^a^ (%)	TSS ^b^ (°Brix)	TSC ^c^ (%)	TCC ^d^ (μg/g)
*C. pentagyna*	3.07 ± 0.07^i^	0.75 ± 0.10^i^	14.99 ± 0.11^f^	7.21 ± 0.43^i^	281.44 ± 6.54^g^
*C. monogyna*	3.93 ± 0.15^c^	0.97 ± 0.04^d^	19.58 ± 0.21^cd^	5.27 ± 0.53^j^	282.74 ± 6.66^g^
*C. pseudomelanocarpa*	3.12 ± 0.13^h^	0.79 ± 0.12^g^	15.65 ± 0.31^f^	10.11 ± 0.44^g^	205.77 ± 4.24^h^
*C. songarica*	3.15 ± 0.16^g^	0.76 ± 0.07^hi^	15.73 ± 0.13^f^	10.91 ± 0.26^g^	141.63 ± 4.13^i^
*C. azarolus* var. *aronia*	3.14 ± 0.10^gh^	1.02 ± 0.15^c^	20.34 ± 0.22^c^	19.43 ± 0.32^a^	359.79 ± 6.15^b^
*C. azarolus* var. *pontica*	3.16 ± 0.17^g^	1.17 ± 0.14^a^	23.43 ± 0.19^a^	15.35 ± 1.23^e^	405.79 ± 5.64^a^
*C. sakranensis*	3.52 ± 0.17^e^	0.89 ± 0.07^ef^	17.59 ± 0.98^de^	19.12 ± 0.86^a^	322.75 ± 1.13^d^
*C. turkestanica*	3.63 ± 0.12^d^	0.91 ± 0.08^e^	18.15 ± 1.21^de^	6.58 ± 0.54^i^	86.84 ± 2.65^j^
*C. meyeri*	3.93 ± 0.14^c^	0.98 ± 0.10^d^	19.65 ± 0.97^c^	19.22 ± 0.55^a^	285.86 ± 5.54^fg^
*C. orientalis*	3.03 ± 0.11^j^	0.77 ± 0.09^ghi^	15.15 ± 0.87^f^	17.55 ± 0.86^b^	340.86 ± 6.76^c^
*C. curvisepala*	4.35 ± 0.10^a^	1.07 ± 0.05^b^	21.75 ± 0.54^b^	8.21 ± 0.45^h^	295.15 ± 7.76^ef^
*C. atrosanguinea*	3.14 ± 0.18^gh^	0.79 ± 0.14^g^	15.67 ± 1.10^f^	15.63 ± 0.75^de^	355.73 ± 2.76^b^
*C. persica*	3.48 ± 0.07^f^	0.87 ± 0.07^f^	17.40 ± 0.58^e^	16.36 ± 0.35^cd^	282.82 ± 5.54^g^
*C. szovitsii*	3.12 ± 0.14^h^	0.78 ± 0.21^gh^	15.59 ± 0.76^f^	17.19 ± 0.87^bc^	366.22 ± 6.76^b^
*C. pseudoheterophylla*	4.03 ± 0.18^b^	1.04 ± 0.09^c^	20.24 ± 0.41^c^	12.61 ± 0.35^f^	316.95 ± 8.23^d^
Significant level	***	***	***	***	***

**Note**: *** Significant at 0.1% level, means with different letters are statistically significant at a 5% level of probability; ^a^ titratable acidity, ^b^ total soluble solids, ^c^ total soluble carbohydrate, ^d^ total carotenoid content.

**Table 4:** Level of total phenols content, total flavonoids, and antioxidant activity in fruits of different hawthorn.

Species	Phytochemical Traits		
	TPC ^a^ (mg GAE/g DW)	TFC ^b^ (mg QUE/g DW)	Antioxidant Activity (mmol Fe^++^/g DW)
*C. pentagyna*	69.12 ± 0.83^a^	5.64 ± 0.14^bc^	1.84 ± 0.21^a^
*C. monogyna*	35.85 ± 0.25^fg^	5.77 ± 0.09^b^	0.93 ± 0.08^e^
*C. pseudomelanocarpa*	65.06 ± 0.67^b^	5.18 ± 0.17^d^	1.10 ± 0.21^d^
*C. songarica*	36.87 ± 0.52^f^	5.47 ± 0.19^c^	0.73 ± 0.12^f^
*C. azarolus* var. *aronia*	27.09 ± 0.63^i^	2.89 ± 0.14^g^	0.79 ± 0.10^ef^
*C. azarolus* var. *pontica*	23.89 ± 0.08^j^	2.74 ± 0.12^g^	0.72 ± 0.06^f^
*C. sakranensis*	31.22 ± 0.11^h^	3.47 ± 0.08^f^	0.51 ± 0.09^gh^
*C. turkestanica*	21.19 ± 0.10^j^	3.21 ± 0.14^f^	0.65 ± 0.03^fg^
*C. meyeri*	58.17 ± 0.82^d^	6.08 ± 0.25^a^	1.27 ± 0.21^c^
*C. orientalis*	40.04 ± 0.25^e^	3.29 ± 0.17^f^	0.45 ± 0.14^hi^
*C. curvisepala*	36.87 ± 0.57^f^	4.29 ± 0.11^e^	0.68 ± 0.12^f^
*C. atrosanguinea*	61.60 ± 0.52^c^	4.37 ± 0.04^e^	1.44 ± 0.21^b^
*C. persica*	31.09 ± 0.88^h^	3.21 ± 0.17^f^	0.32 ± 0.18^i^
*C. szovitsii*	33.50 ± 0.33^gh^	2.44 ± 0.12^h^	0.66 ± 0.08^fg^
*C. pseudoheterophylla*	59.25 ± 0.64^cd^	4.42 ± 0.28^e^	1.12 ± 0.18^cd^
Significant level	***	***	***

**Note**: *** Significant at 0.1% level, means with different letters are statistically significant at a 5% level of probability. ^a^ Total phenol content, ^b^ total flavonoid content. GAE: gallic acid equivalent; QUE: quercetin equivalent, DW: dry weight.

**Table 5:** Content of phenolic compounds in fruits of different hawthorn (*Crataegus* spp.) species.

Fruit Phenolic Compounds (mg/g DW)							
Species	Chlorogenic acid	Vitexin 2-*O*-rhamnoside	Vitexin	Rutin	Hyperoside	Isoquercetin	Quercetin
*C. pentagyna*	0.50 ± 0.06^de^	-	0.07 ± 0.02^e-g^	1.27 ± 0.08^b^	2.41 ± 0.04^b^	1.18 ± 0.07^b^	0.04 ± 0.00^a^
*C. monogyna*	0.40 ± 0.04^ef^	-	0.18 ± 0.05^bc^	-	1.15 ± 0.07^i^	0.68 ± 0.04^d-f^	0.05 ± 0.00^a^
*C. pseudomelanocarpa*	1.16 ± 0.06^a^	-	0.15 ± 0.04^b-e^	2.68 ± 0.07^a^	2.11 ± 0.06^cd^	1.56 ± 0.07^a^	0.04 ± 0.01^a^
*C. songarica*	0.94 ± 0.03^b^	-	0.13 ± 0.05^c-f^	0.24 ± 0.05^de^	1.74 ± 0.03^e^	0.76 ± 0.08^d^	0.05 ± 0.01^a^
*C. azarolus* var. *aronia*	0.51 ± 0.05^d^	-	0.09 ± 0.01^d-f^	0.15 ± 0.04^fg^	1.05 ± 0.07^i^	0.59 ± 0.06^ef^	0.05 ± 0.02^a^
*C. azarolus* var. *pontica*	0.71 ± 0.04^c^	-	0.11 ± 0.00 ^c-f^	0.16 ± 0.07^e-g^	1.07 ± 0.05^i^	0.61 ± 0.04^ef^	0.05 ± 0.02^a^
*C. sakranensis*	0.03 ± 0.02^i^	-	0.06 ± 0.02^fg^	-	-	0.24 ± 0.08^g^	0.03 ± 0.02^a^
*C. turkestanica*	0.40 ± 0.05^ef^	-	0.16 ± 0.07^b-d^	0.21 ± 0.07^d-f^	1.50 ± 0.08^g^	0.58 ± 0.05^f^	0.05 ± 0.01^a^
*C. meyeri*	0.06 ± 0.00^i^	-	0.08 ± 0.06^d-g^	0.28 ± 0.08^d^	2.94 ± 0.02^a^	1.59 ± 0.06^a^	0.06 ± 0.01^a^
*C. orientalis*	0.77 ± 0.06^c^	-	-	0.39 ± 0.03^c^	1.62 ± 0.04^f^	0.78 ± 0.08^d^	0.03 ± 0.02^a^
*C. curvisepala*	1.04 ± 0.05^b^	0.08 ± 0.02^b^	0.22 ± 0.05^b^	0.24 ± 0.03^de^	2.21 ± 0.05^c^	0.65 ± 0.06^ef^	0.05± 0.02^a^
*C. atrosanguinea*	0.51 ± 0.03^d^	-	0.19 ± 0.07^bc^	0.23 ± 0.04^d-f^	2.51 ± 0.03^b^	1.22 ± 0.03^b^	0.05 ± 0.02^a^
*C. persica*	0.44 ± 0.06^ef^	-	0.08 ± 0.09^d-g^	0.12 ± 0.07^g^	1.32 ± 0.08^h^	0.70 ± 0.05^de^	0.04 ± 0.01^a^
*C. szovitsii*	0.39 ± 0.08^fg^	-	0.31 ± 0.06^a^	0.38 ± 0.06^c^	0.87 ± 0.09^j^	0.34 ± 0.07^g^	0.04 ± 0.02^a^
*C. pseudoheterophylla*	0.32 ± 0.04^fg^	0.17 ± 0.09^a^	0.07 ± 0.03^e-g^	0.23 ± 0.03^d-f^	2.05 ± 0.05^d^	0.98 ± 0.04^c^	0.04 ± 0.01^a^
Significant level	***	***	***	***	***	***	ns

**Note:** *** and ns, Significant at 0.1% level and not significant, respectively, means with different letters are statistically significant at a 5% level of probability.
